# Renal Solitary Fibrous Tumor With Local Recurrence Following Complete Surgical Resection: A Case Report

**DOI:** 10.7759/cureus.104123

**Published:** 2026-02-23

**Authors:** Panagiotis A Panagopoulos, Konstantinos Douroumis, Thomas Kalfas, Penelope Korkolopoulou, Eirini Thymara, Maria Papanikolaou, Konstantinos Belogiannis, Napoleon Moulavasilis, Evangelos Fragkiadis, Konstandinos Stravodimos

**Affiliations:** 1 Urology, National and Kapodistrian University of Athens School of Medicine, Athens, GRC; 2 First Department of Pathology, National and Kapodistrian University of Athens School of Medicine, Athens, GRC

**Keywords:** local recurrence, mesenchymal neoplasm, pleura, renal neoplasms, solitary fibrous tumor

## Abstract

Solitary fibrous tumor (SFT) is a rare mesenchymal neoplasm that most commonly arises in the pleura. Renal SFT is extremely uncommon, and local recurrence after complete surgical resection is rarely reported. In our case, an 81-year-old woman underwent laparoscopic radical nephrectomy for an incidentally detected left renal mass. Histopathological and immunohistochemical analysis confirmed a malignant SFT, with STAT6 and CD34 positivity, and classified it as intermediate risk according to the modified Demicco model. Despite negative imaging at six months, local recurrence was detected one year postoperatively and confirmed by biopsy. Renal SFTs may demonstrate unpredictable biological behavior, including local recurrence after apparently complete resection. Close and long-term follow-up is essential for optimal patient management.

## Introduction

Solitary fibrous tumor (SFT) is a rare subtype of spindle-cell mesenchymal tumor and was first reported by Wagner in 1870. It is most commonly located within the pleural cavity; however, 30% of cases are found outside the pleura [1]. SFT typically affects adults, with peak incidence between 40 and 70 years of age, and shows no sex predilection [2]. It more frequently arises in deep soft tissue. As with other sarcomatoid tumors, surgical resection is the standard of care, since the tumor grows slowly and generally has a favorable prognosis. On immunohistochemistry, SFT is usually positive for CD34 and STAT6 [3].

The Demicco template is a standard tool used to evaluate the metastatic risk of SFT [4]. The original Demicco system includes three variables: patient age, tumor size, and mitotic count. An alternative version includes a fourth variable - the presence of necrosis - which is also widely used [5].

SFT of the kidney is an extremely rare tumor, very unlikely to locally or distantly recur. Renal SFTs are typically considered as renal cell carcinoma on imaging studies, given the lack of pathognomonic radiological findings [6]. To our knowledge, local recurrence of SFT has been reported in the literature in very few cases [7-9].

We report a case of local recurrence of an SFT following laparoscopic radical nephrectomy, with the aim of contributing to the existing literature on this rare entity.

## Case presentation

**Figure 1:**
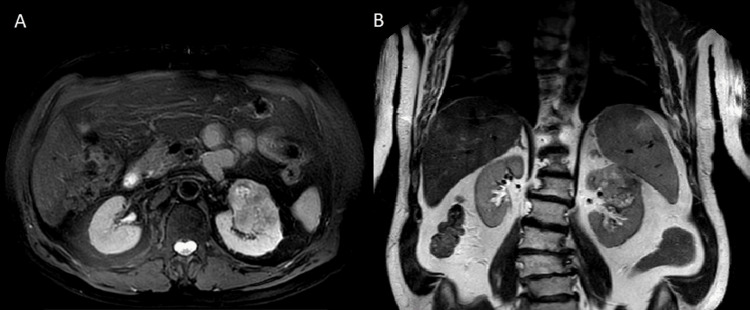
(A) T2 fat suppression axial and (B) T2 coronal A solid mass in the upper pole of the left kidney

An 81-year-old female patient was referred to the outpatient department of our clinic due to a left renal mass, incidentally detected by ultrasonography, which had been performed during a routine health check-up. Clinical investigation, blood and urine analysis were unremarkable. Subsequent imaging examinations were ordered. MRI scan revealed a heterogeneous mass at the upper pole of the left kidney, which measured 6.4x5x5.2 cm with relatively ill-defined margins, showing intermediate signal intensity on T2-weighted imaging (Figure 1). The chest CT scan was negative for distant metastasis. The patient underwent a laparoscopic left radical nephrectomy and was discharged on the third postoperative day.

**Figure 2:**
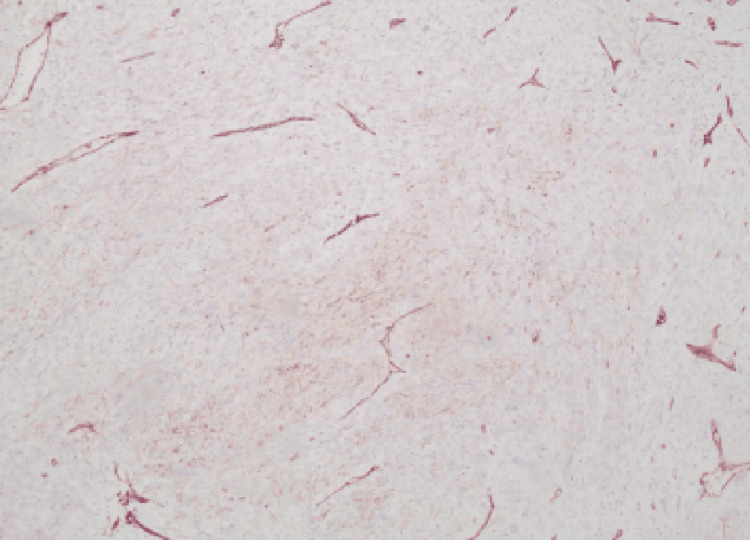
Positive CD34 stain (x100 magnification) CD34 normally stains endothelial cells and has high sensitivity (95%) but low specificity for SFT. SFT, solitary fibrous tumor.

**Figure 3:**
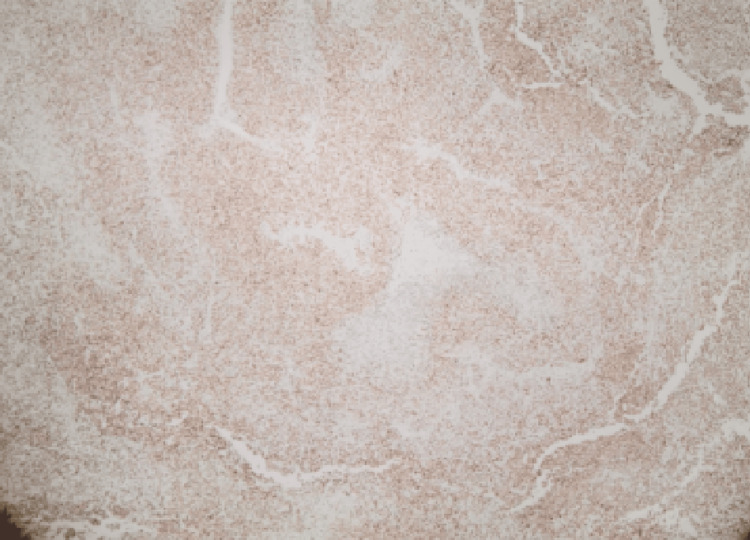
Positive STAT6 stain (x40 magnification) STAT6 is considered the most sensitive and specific immunohistochemical marker for SFT because most SFTs contain a *NAB2-STAT6* fusion gene.

Macroscopically, the tumor consisted of a whitish solid mass measuring 6.4 cm in diameter. Microscopic examination revealed that the tumor was composed of oval and spindle-shaped cells with moderate to severe cellular atypia, along with the presence of scattered giant cells. It can be identified as mitotic atypia (>20/10 high-power field (HPF)). The neoplasm exhibited extensive necrosis (over 40%) and infiltrated the venous vessels of the renal pelvis, without extending into the perinephric fat. Immunohistochemical staining revealed STAT6 and CD34. GATA-3 was weakly positive (Figures 2, 3). Surgical margins were negative for malignancy. Based on the above findings, the pathology report concluded that the malignancy was an SFT.

Using the modified four-variable risk stratification model for the development of metastasis in SFTs, which takes into account age (>55 years), tumor size (5-10 cm), mitotic activity (≥24/HPF), and tumor necrosis (≥10%), the patient is classified as intermediate risk [5]. The multidisciplinary tumor board, consisting of urologists, oncologists, and radiation oncologists at our hospital, suggested close follow-up every six months.

**Figure 4:**
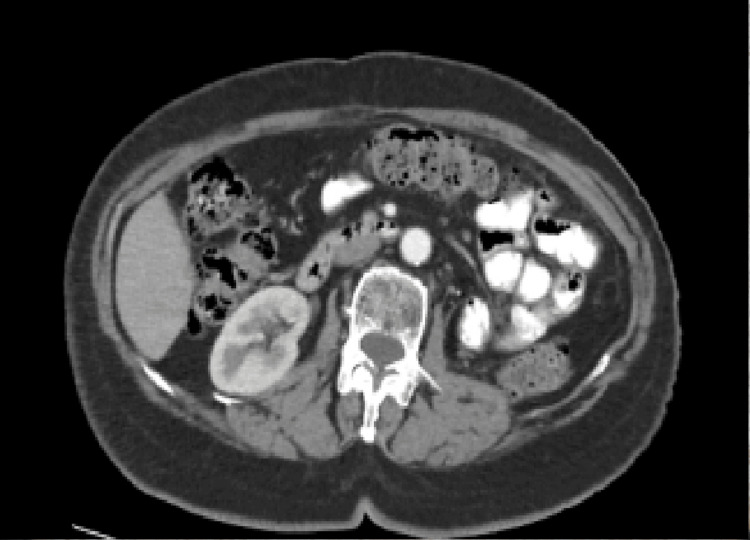
CT scan with IV contrast at six months after operation Six months after surgery, the CT scan was normal, and no site of recurrence was observed.

The first follow-up CT scan, performed six months after surgery, showed no detectable mass in the left renal space or any other organ (Figure 4).

**Figure 5:**
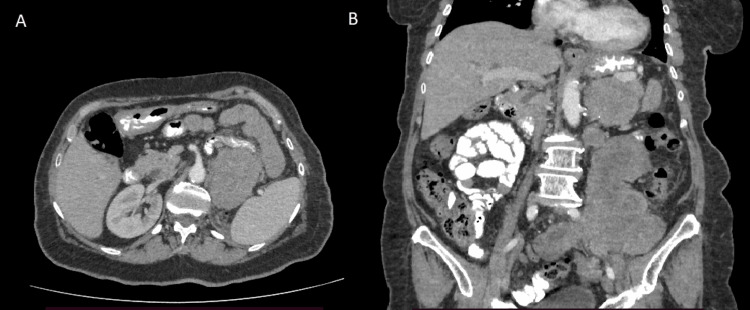
(A) Axial and (B) coronal imaging of recurrent renal solitary tumor one year after operation A big solid mass recurrence one year after surgery arising from the left renal space to the pelvis.

One year after the operation, the patient complained of left lumbar pain and mild abdominal pain. Clinical examination revealed a palpable mass in the left abdominal and lumbar region. An abdominal CT scan was performed and revealed a 13x10 mass lesion in the retroperitoneal area, and a 7.3x2.7 cm lesion in the left iliac fossa (Figure 5). The patient underwent a percutaneous biopsy. The biopsy cores were compatible with a neoplasm, with immunomorphological findings similar to those of the primary tumor (STAT6 and CD34 were positive). All the above findings were compatible with local recurrence from the SFT.

The multidisciplinary tumor board at our hospital, taking into account the patient’s wish to not proceed with any further treatment and the limited therapeutic options for SFTs, offered palliative care only.

## Discussion

SFTs may occur at any site, but they have been mostly observed in the pleura. SFT of the kidney is a rare entity [10]. Local recurrence is even rarer, as only three cases of local recurrence have been reported in the literature (Table 1) [7-9]. Our patient presented with a left renal mass discovered incidentally during a routine examination. Histopathology revealed spindle cells with atypia, scattered giant cells, and extensive necrosis (>40%), along with vascular invasion features consistent with malignant SFT.

**Table 1:** Age, size, mitotic count, and time to recurrence Summary table comparing three other cases of local recurrence. HPF, high-power field; SFT, solitary fibrous tumor.

Study	Age (years)	Primary tumor size (cm)	Mitotic count (per 10 HPF)	Time to recurrence
Sammoud et al. (malignant renal SFT with two local recurrences) [9]	53	13×10×8	7/10 HPF	First recurrence at 6 months and second recurrence at 9 months
Cheung et al. (SFT of the kidney developing local recurrence) [11]	50	17×11×8	Not clearly quantified, and low mitotic activity reported	36 months
Sfoungaristos et al. (Massive retroperitoneal recurrence) [7]	46	9 cm	Not specified; Ki-67 2%-7%	6 months

Immunohistochemistry confirmed the diagnosis with nuclear STAT6 and CD34 positivity. CD34 has been reported to be diffused in many cases of SFTs, and it is currently the most useful tool for recognizing SFTs, regardless of the fact that it cannot be considered pathognomonic for diagnosis [12].

According to the modified Demicco risk model, this patient was categorized as intermediate risk, which correlated with her subsequent clinical course. Local recurrence occurred one year after nephrectomy, despite complete resection and negative imaging at six months. This emphasizes the unpredictable biological behavior of SFT and raises strong concerns regarding the optimal follow-up.

Surgical resection is considered to be the standard of care with a favorable prognosis for renal SFTs [13]. In our case, given the large recurrence (13×10 cm) detected only six months after the previous CT scan, concerns were raised regarding a possible sampling error or intraoperative tumor seeding. The pathological specimens were re-examined, and the findings remained unchanged. Regarding the possibility of tumor seeding during surgery, no intraoperative events of concern were noted to our knowledge that would suggest cancer dissemination. Most of these renal tumors in the literature were benign, and only a few of them revealed malignant features [11]. Approximately 10%-15% of intrathoracic SFTs and around 10% of extrathoracic SFTs are expected to recur or metastasize. The prognosis of SFT may be unfavorable in cases of incomplete surgical excision, large tumor size (>10 cm), or when the tumor arises at an extrathoracic site [12]. For this reason, SFT is classified as an intermediate-grade neoplasm with infrequent metastatic potential. The use of chemotherapy and antiangiogenic agents has been described only in the setting of metastatic disease [14]. Currently, there are no established indications or standardized chemotherapy protocols for SFT. Agents typically used for soft tissue sarcomas have shown some relative efficacy, although SFTs are generally regarded as resistant to systemic chemotherapy [14]. In our case, the multidisciplinary tumor board, taking into account and respecting the patient’s wishes, decided not to proceed with any further systemic treatment and recommended palliative care only.

## Conclusions

Renal SFTs represent an exceptionally rare clinical entity, often indistinguishable from renal cell carcinoma on imaging and usually diagnosed only after surgical excision. Although most reported renal SFTs exhibit benign behavior, malignant features and local recurrence, as demonstrated in this case, underscore the unpredictable biological course of these tumors. Our patient developed an early local recurrence one year after complete surgical resection with negative margins, despite being classified as intermediate risk according to the modified Demicco model. This highlights the limitations of current risk stratification systems and emphasizes the need for vigilant, long-term follow-up, even in cases without high-risk features. Histopathological evaluation combined with immunohistochemical confirmation, particularly STAT6 and CD34 positivity, remains essential for accurate diagnosis. Given the absence of standardized systemic therapies and the limited efficacy of chemotherapy in SFTs, surgical resection remains the cornerstone of treatment, while management of recurrence should be individualized through a multidisciplinary approach. Reporting rare cases of renal SFT with malignant behavior and recurrence is crucial to improving understanding of their natural history and to refining follow-up strategies and therapeutic decision-making in the future.

## References

[1] Myoteri D, Dellaportas D, Nastos C, Gioti I, Gkiokas G, Carvounis E, Theodosopoulos T (2017). Retroperitoneal solitary fibrous tumor: a "patternless" tumor. Case Rep Oncol Med.

[2] Sugita S, Segawa K, Kikuchi N (2022). Prognostic usefulness of a modified risk model for solitary fibrous tumor that includes the Ki-67 labeling index. World J Surg Oncol.

[3] Sbaraglia M, Bellan E, Dei Tos AP (2020). The 2020 WHO classification of soft tissue tumours: news and perspectives. Pathologica.

[4] Demicco EG, Park MS, Araujo DM (2012). Solitary fibrous tumor: a clinicopathological study of 110 cases and proposed risk assessment model. Mod Pathol.

[5] Demicco EG, Wagner MJ, Maki RG, Gupta V, Iofin I, Lazar AJ, Wang WL (2017). Risk assessment in solitary fibrous tumors: validation and refinement of a risk stratification model. Mod Pathol.

[6] Zaghbib S, Chakroun M, Essid MA (2019). Solitary fibrous tumor of the kidney: a case report. Int J Surg Case Rep.

[7] Sfoungaristos S, Papatheodorou M, Kavouras A, Perimenis P (2012). Solitary fibrous tumor of the kidney with massive retroperitoneal recurrence. A case presentation. Prague Med Rep.

[8] Usuba W, Sasaki H, Yoshie H (2016). Solitary fibrous tumor of the kidney developing local recurrence. Case Rep Urol.

[9] Sammoud S, Ferjani S, Hamdani M, Toumi A (2019). Malignant renal solitary fibrous tumor with two local recurrences and distant pulmonary metastasis. Urology.

[10] Constantinidis C, Koutalellis G, Liapis G, Stravodimos C, Alexandrou P, Adamakis I (2007). A solitary fibrous tumor of the kidney in a 26-year-old man. Can J Urol.

[11] Cheung F, Talanki VR, Liu J, Davis JE, Waltzer WC, Corcoran AT (2016). Metachronous malignant solitary fibrous tumor of kidney: case report and review of literature. Urol Case Rep.

[12] Zhao G, Li G, Han R (2012). Two malignant solitary fibrous tumors in one kidney: case report and review of the literature. Oncol Lett.

[13] Morimitsu Y, Nakajima M, Hisaoka M, Hashimoto H (2000). Extrapleural solitary fibrous tumor: clinicopathologic study of 17 cases and molecular analysis of the p53 pathway. APMIS.

[14] Grobmyer SR, Maki RG, Demetri GD, Mazumdar M, Riedel E, Brennan MF, Singer S (2004). Neo-adjuvant chemotherapy for primary high-grade extremity soft tissue sarcoma. Ann Oncol.

